# Endoplasmic reticulum–localized UBC34 interaction with lignin repressors MYB221 and MYB156 regulates the transactivity of the transcription factors in *Populus tomentosa*

**DOI:** 10.1186/s12870-019-1697-y

**Published:** 2019-03-12

**Authors:** Lin Zheng, Yajuan Chen, Dong Ding, Ying Zhou, Liping Ding, Jianhua Wei, Hongzhi Wang

**Affiliations:** 1 Beijing Agro-Biotechnology Research Center, Beijing Academy of Agricultural and Forestry Sciences, No. 9, Shuguang Huayuan Middle Road, Haidian District, Beijing, 100097 People’s Republic of China; 2 Beijing Key Laboratory of Agricultural Genetic Resources and Biotechnology, Beijing Academy of Agricultural and Forestry Sciences, No. 9, Shuguang Huayuan Middle Road, Haidian District, Beijing, 100097 People’s Republic of China

**Keywords:** Lignin biosynthesis, PtoUBC34, Transcriptional repressor, PtoMYB221, PtoMYB156, ER-associated degradation

## Abstract

**Background:**

Regulation of lignin biosynthesis is known to occur at the level of transcription factors (TFs), of which R2R3-MYB family members have been proposed to play a central role via the AC *cis*-elements. Despite the important roles of TFs in lignin biosynthesis, the post-translational regulation of these TFs, particularly their ubiquitination regulation, has not been thoroughly explored.

**Results:**

We describe the discovery of a *Populus tomentosa* E2 ubiquitin-conjugating enzyme 34 (PtoUBC34), which is involved in the post-translational regulation of transactivation activity of lignin-associated transcriptional repressors PtoMYB221 and PtoMYB156. PtoUBC34 is localized at the endoplasmic reticulum (ER) membrane where it interacts with transcriptional repressors PtoMYB221 and PtoMYB156. This specific interaction allows for the translocation of TFs PtoMYB221 and PtoMYB156 to the ER and reduces their repression activity in a *PtoUBC34* abundance-dependent manner. By taking a molecular biology approach with quantitative real-time polymerase chain reaction (qRT-PCR) analysis, we found that *PtoUBC34* is expressed in all aboveground tissues of trees in *P. tomentosa*, and in particular, it is ubiquitous in all distinct differentiation stages across wood formation, including phloem differentiation, cambium maintaining, early and developing xylem differentiation, secondary cell wall thickening, and programmed cell death. Additionally, we discovered that *PtoUBC34* is induced by treatment with sodium chloride and heat shock.

**Conclusions:**

Our data suggest a possible mechanism by which lignin biosynthesis is regulated by ER-localized PtoUBC34 in poplar, probably through the ER-associated degradation (ERAD) of lignin-associated repressors PtoMYB221 and PtoMYB156.

**Electronic supplementary material:**

The online version of this article (10.1186/s12870-019-1697-y) contains supplementary material, which is available to authorized users.

## Background

Lignin is an aromatic heteropolymer that deposits mostly in the secondary cell walls of tracheophytes and helps large upright land plants to survive by providing mechanical support and efficient water conductance [1]. It is the second most abundant polymer in wood and has been estimated to represent almost 30% of the total biomass produced in the biosphere [2]. However, lignin limits the efficient utilization of lignocellulose as feedstock in pulping, papermaking, and biofuel industry, in which lignin removal is a necessary and costly step [3–5]. A preferred strategy to optimize lignocellulosic feedstock with improved performance in various applications is to utilize biotechnology to engineer the desired amount and structure of lignin [5–7]. Elucidation of the mechanisms underlying lignin biosynthesis and regulation is critical in plant biology and likely will guide the lignin bioengineering design.

For more than 20 years, the lignin biosynthesis field has been focused on identifying the monolignol biosynthetic genes and their transcriptional regulators. Many steps of the biochemical pathway and transcriptional control of the monolignol biosynthesis already have been discovered [2, 8–10]. It is well known that regulation of lignin biosynthesis occurs at the level of transcription factors (TFs) [8–10]. Among these, R2R3-MYB TFs are key players [11–14] which coordinate the gene expression of the complex monolignol biosynthetic pathway by binding to the AC *cis*-elements present in the promoter regions of most monolignol biosynthetic genes [15–18]. The identified R2R3-MYB key players in lignin biosynthesis include secondary cell wall master activators AtMYB46 and AtMY83 from *Arabidopsis*, and their orthologs in other plant species [19–26]; and lignin-specific activators MYB58, MYB63, MYB85 in *Arabidopsis*, and PtMYB1 in pine [17, 18, 27, 28]; and lignin transcriptional repressors, most of which belong to subgroup 4 of the R2R3-MYB TF family [29–37] (Table 1). The unique TF-directed hierarchical gene regulatory networks [38] in lignin biosynthesis integrate transcriptional activators and repressors to maintain transcriptional homeostasis of lignin pathway genes [8, 13]. Failure to maintain the transcriptional homeostasis results in limited growth and ectopic lignin deposition, as demonstrated by overexpression of the MYB lignin activators AtMYB46, AtMYB85, AtMYB58, and AtMYB63 [17, 19, 27]. Indeed, transcriptional repression through repressors is a useful strategy used to maintain transcriptional homeostasis [39].

**Table 1:** Key R2R3-MYB regulators identified in the transcriptional network of lignin biosynthesis in plants

Protein name	ID	Plant	Function and references
Secondary cell wall master activators			
AtMYB46	At5g12870	*Arabidopsis thaliana*	Regulates the biosynthesis of cellulose, hemicellulose and lignin of secondary walls [19–21]
AtMYB83	At3g08500	*Arabidopsis thaliana*	Act redundantly with AtMYB46 [22]
PtrMYB3	Potri.001G267300	*Populus trichocarpa*	Regulates the biosynthesis of cellulose, hemicellulose and lignin of secondary walls [23]
PtrMYB20	Potri.009G061500	*Populus trichocarpa*	Regulates the biosynthesis of cellulose, hemicellulose and lignin of secondary walls [23]
PtMYB4	AY356371	*Pinus taeda*	Regulates lignin biosynthesis [24]
EgMYB2	AJ576023	*Eucalyptus gunnii*	Regulates secondary wall formation [25]
OsMYB46	Os12g0515300	*Oryza sativa*	Regulates the biosynthesis of cellulose, hemicellulose and lignin of secondary walls [26]
ZmMYB46	JN634085	*Zea mays*	Regulates the biosynthesis of cellulose, hemicellulose and lignin of secondary walls [26]
Lignin specific activators			
AtMYB58	At1g16490	*Arabidopsis thaliana*	Activates lignin biosynthetic pathway [17]
AtMYB63	At1g79180	*Arabidopsis thaliana*	Activates lignin biosynthetic pathway [17]
AtMYB85	At4g22680	*Arabidopsis thaliana*	Activates lignin biosynthetic pathway [27]
PtMYB1	AY356372	*Pinus taeda*	Regulates lignin biosynthesis [18, 28]
Lignin transcriptional repressors			
AtMYB4	At4g38620	*Arabidopsis thaliana*	A repressor from subgroup 4 of R2R3-MYB family, represses SND1 and lignin biosynthesis [29]
AtMYB32	At4g34990	*Arabidopsis thaliana*	A repressor from subgroup 4 of R2R3-MYB family, represses SND1 and lignin biosynthesis [29]
PvMYB4	JF299185	*Panicum virgatum*	A repressor from subgroup 4 of R2R3-MYB family, down-regulates lignin biosynthesis [30]
ZmMYB31	NM_001112479	*Zea mays*	A repressor from subgroup 4 of R2R3-MYB family, represses lignin biosynthesis [31, 32]
ZmMYB42	NM_001112539	*Zea mays*	A repressor from subgroup 4 of R2R3-MYB family, represses lignin biosynthesis [33]
PttMYB21a	AJ567345	*Populus tremula x Populus tremuloides*	Negatively regulates lignin biosynthesis [34]
TaMYB4	JF746995	*Triticum aestivum*	A repressor from subgroup 4 of R2R3-MYB family, negatively regulates lignin biosynthesis [35]
PdMYB221	not available	*Populus deltoids*	A repressor from subgroup 4 of R2R3-MYB family, negatively regulates the secondary wall formation [36]
PtoMYB156	KT990214	*Populus tomentosa*	A repressor from subgroup 4 of R2R3-MYB family, represses phenylpropanoid biosynthesis [37]

Although lignin biosynthesis and transcriptional regulation have been studied extensively, the post-translational regulation of lignin complex biosynthetic pathway, particularly ubiquitination, was not studied in detail until recent years. Ubiquitination of a substrate, which has been shown to play crucial roles in plant senescence, xylogenesis, embryogenesis, floral development, and stress responses [40–42], is performed by a set of enzymes: ubiquitin activation enzyme (E1), ubiquitin-conjugating enzyme (E2), and ubiquitin ligase (E3) [43]. In *Arabidopsis*, a WD40-repeat adaptor protein AtULCS1, a subunit of a cullin-RING E3 ligase, was shown to function in cell wall modification and lignin-specific deposition in anther cell wall, and control anther dehiscence [44]. In rice, an F-box protein OsFBK1, a component of an SCF E3 ligase, has been shown to play crucial roles in the site-specific deposition of lignin in the anther endothecium, which mediates the turnover of a cinnamoyl-CoA reductase. Importantly, this specific lignin deposition is believed to regulate anther dehiscence and plant fertility [45]. The ubiquitination regulation of lignin deposition in poplar has not yet been reported, even though lignin accounts for approximately 25% of the total lignocellulose in poplar.

Given the crucial roles that lignin repressors play in maintaining transcriptional homeostasis of complex lignin biosynthetic pathway [39] and activating lignin deposition in plant defense [8] and because their conditional expression responds to the developmental signals and environmental cues [32, 33, 46], we propose that fine-tuned mechanisms post-translationally regulate transactivation activity of subgroup 4 MYB lignin repressors MYB221 and MYB156 in poplar, which likely mediate the regulation of secondary wall formation and lignin biosynthesis. Because protein-protein interactions are a common strategy to modify the activity of TFs post-translationally [47], it is essential to isolate proteins that interact with poplar subgroup 4 MYB221 and MYB156 repressors.

In this study, we screened *P. tomentosa* differentiating secondary vascular tissue cDNA library with PtoMYB221 and PtoMYB156 as baits, and isolated a partial sequence of PtoUBC34, a specific ubiquitin-conjugating enzyme. Further characterization through co-localization, translocation, bimolecular fluorescence complementation (BiFC), coimmunoprecipitation, and transactivation assay in poplar mesophyll protoplasts suggested that PtoUBC34 was involved in the lignin biosynthesis through the regulation of transactivity of lignin transcriptional repressors, probably by the ER-associated degradation (ERAD) pathway in *P. tomentosa*.

## Results

### Isolation of proteins that interact with PtoMYB221 and PtoMYB156

**Fig. 1:**
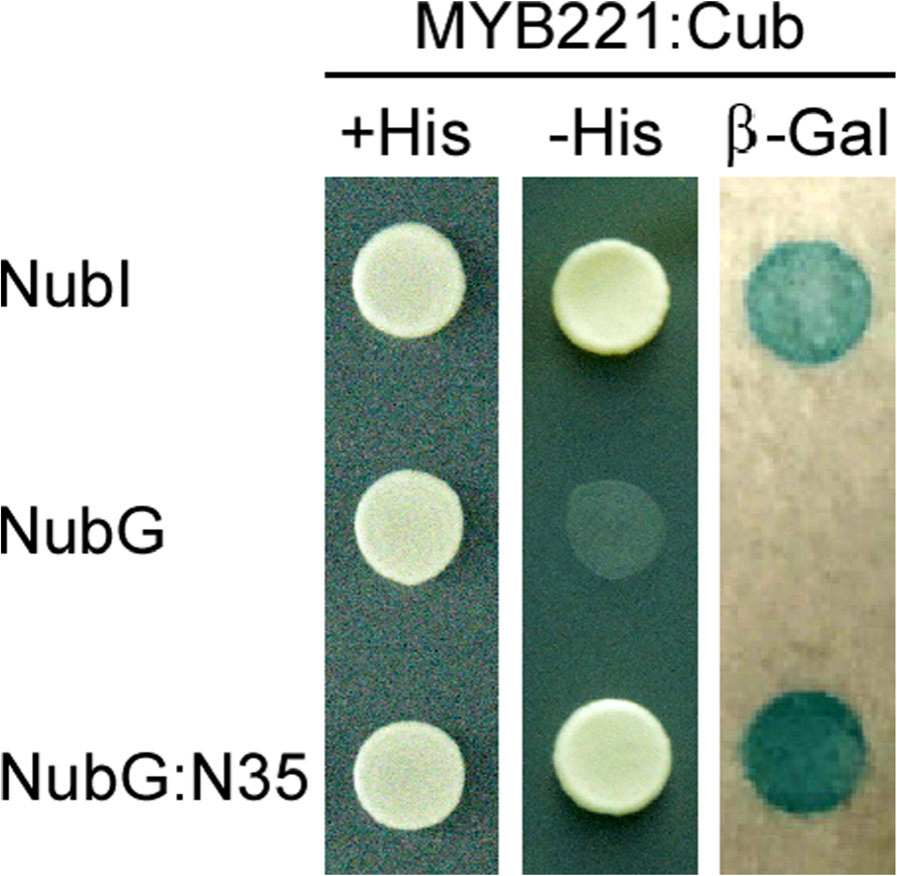
The specific interaction between PtoMYB221 and the N35 protein in a split-ubiquitin Y2H system. Bait protein PtoMYB221 fused to Cub was co-expressed in yeast NMY51 with prey proteins, NubI, NubG, or the NubG:N35 protein. NubI is a positive prey control with strong affinity between wild-type NubI and Cub, whereas NubG is a negative prey control with weak affinity between mutant NubG and Cub. Interaction of the bait was shown with positive control NubI and the test NubG:N35 fusion protein but not with the negative control NubG, as indicated by growth of the transformed strain on synthetic defined medium without Trp, Leu, and His and by the activity of β-Gal

To isolate proteins that potentially interact with MYB subgroup 4 transcriptional repressors PtoMYB221 and PtoMYB156 during wood formation, we used the yeast two-hybridization (Y2H) system based on split-ubiquitin to screen normalized cDNA library from *P. tomentosa* differentiating secondary vascular tissue (including vascular cambium, developing phloem, and xylem). In this library vector, N-terminal half of the ubiquitin (Nub) is modified at position 3 of the protein, where isoleucine is exchanged with glycine (NubI → NubG), to abolish the strong affinity between wild-type Nub and C-terminal half of the ubiquitin (Cub). This system can limit self-activation by TF proteins in the classical protein interaction assay [48], in which transcriptional repressor PtoMYB156 has been shown to significantly activate the transcription of *his3* and β-galactosidase (*β-Gal*) reporter genes (Additional file 1: Figure S1). To prevent leaky expression of the *his3* reporter gene, we added 3-aminotriazole (1 mM) to the selection medium. We fused the entire PtoMYB221 or PtoMYB156 protein to the Cub and used them as baits. We transformed 28 μg of library DNA into yeast strain NMY51 containing PtoMYB221 or PtoMYB156 bait vector as recommended in the user manual. We rescued approximately 5 × 10^6^ independent transformants, covering the cDNA library about 2.5 times, whose complexity is 1.92 × 10^6^ independent clones. We screened the transformants by growth selection in medium lacking histidine and then by the activity of β-Gal. A protein designated N35 was isolated and was capable of activating transcription of the two reporter genes in the presence of PtoMYB221. We confirmed the specific interaction between N35 and PtoMYB221 by the re-introduction of the corresponding plasmids into yeast NMY51 (Fig. 1).

### N35 represents a partial sequence of an ER-localized E2 ubiquitin-conjugating enzyme

**Fig. 2:**
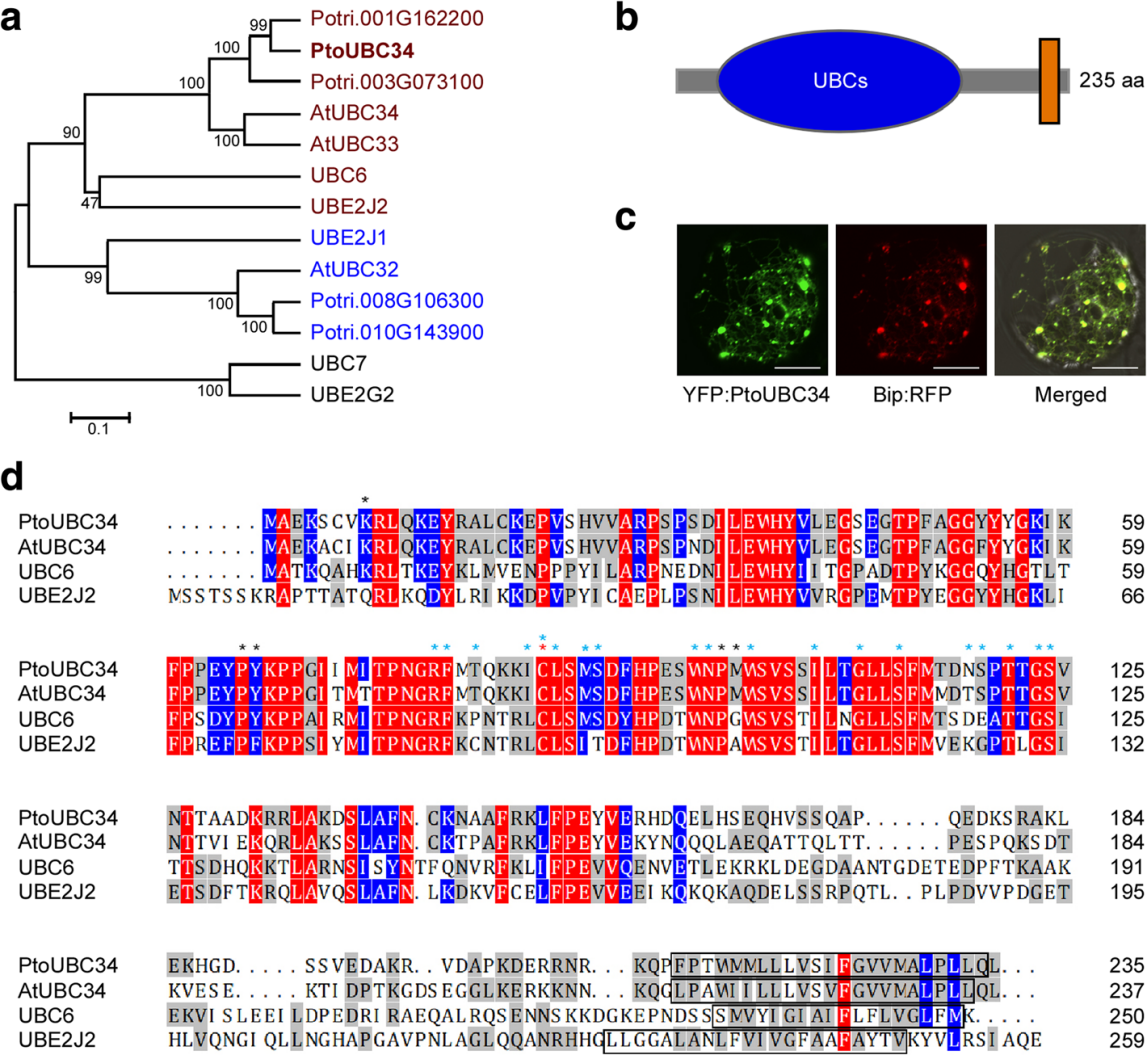
Characteristics of PtoUBC34. **a.** Phylogenetic analysis of PtoUBC34 and other ERAD-related UBC proteins in yeast (UBC6 and UBC7), humans (UBE2J1, UBE2J2, and UBE2G2), poplar, and *Arabidopsis*. The phylogenetic tree was established with full-length protein sequences by the neighbor-joining method. The bootstra*p* values out of 500 retrials are indicated at each branch. The scale (0.1) represents a 10% change in sequences. **b.** Schematic of PtoUBC34. The UBCc domain (blue) and a transmembrane domain (orange) are predicted in PtoUBC34 at the N-terminus and the C-terminus, respectively. **c.** Subcellular localizatin of PtoUBC34. *PtoUBC34* was fused with *YFP* and co-transformed with the ER marker *BiP:RFP* [57] into *P. tomentosa* protoplasts*.* **d.** Multiple sequence alignment of yeast UBC6, human UBE2J2, *Arabidopsis* UBC34 and *P. tomentosa* UBC34. The conserved active Cys residue with red star, E3 interaction residues with black stars and Ub thioester intermediate interaction residues with blue stars are indicated. The transmembrane regions are shown in boxes. Bar in (c) = 10 μm

Sequence analysis revealed that N35 is a partial sequence of an E2 ubiquitin-conjugating enzyme using a BLASTP search against the *P. trichocarpa* genome (http://phytozome.jgi.doe.gov/pz/portal.html), with the best hit for Potri.001G162200. The corresponding coding sequence (CDS) of the N35 protein was missing the first 72 nucleotides. Then we obtained the full-length cDNA sequence from *P. tomentosa* by reverse transcription polymerase chain reaction (RT-PCR) with primers designed according to the homologous sequence of Potri.001G162200 and deposited the sequence into the GenBank with accession number MH708242. It shares 98.7 and 98.30% sequence identity with orthologs from *P. trichocarpa* (Potri.001G162200) and *Populus euphratica* (PeUBC34-like) in amino acid, respectively, and was named after PtoUBC34. In comparison, the truncated UBC34 of the N35 protein was designated as PtoUBC34s. PtoUBC34 is homologous with the yeast Ubc6 and mammalian UBE2J1 and UBE2J2, which are involved in the ERAD process [49–52]. A whole-genome BLAST search (https://phytozome.jgi.doe.gov) indicated that three other genes are homologous with Potri.001G162200 in *P. trichocarpa*: Potri.003G073100, Potri.008G106300 and Potri.010G143900. All four genes are homologous with yeast Ubc6 and mammalian UBE2J1 and UBE2J2. Similarly to *Arabidopsis* and humans [52–55], *P. trichocarpa* Ubc6 homologs evolved into two subfamilies, and Potri.001G162200 and Potri.003G073100 are both similar to UBE2J2, whereas Potri.008G106300 and Potri.010G143900 belong to the UBE2J1 subfamily (Fig. 2a).

*PtoUBC34* encodes a protein composed of 235 amino acid with a predicted molecular mass of ~ 27 kDa using Compute pI/Mw (https://web.expasy.org/compute_pi/). InterPro program (http://www.ebi.ac.uk/interpro/) [56] predicted the presence of an E2 ubiqutin-conjugating enzyme catalytic (UBCc) domain located at the N-terminus and a transmembrane domain at the C-terminus of the PtoUBC34 protein. The UBCc domain includes a conserved active Cys at position 87, which forms a thiol-ester with ubiquitin and serves as a point of attachment for ubiquitin before transfer to the cellular target, and several E3 interaction residues 8 K, 65P, 66Y, 100P, 101 M, and many Ub thioester intermediate interaction residues 79R, 80F, 82 T, 86I, 87C, 88 L, 90 M, 91S, 98 W, 99 N, 102 W, 107I, 110G, 113S, 118 N, 119S, 121 T, 123G, and 124S, most of which are well conserved among yeast, humans, and plants (Fig. 2b and d). To investigate the subcellular localization of the PtoUBC34 protein, the yellow fluorescent protein (YFP)-UBC34 fusion protein (YFP:PtoUBC34) driven by the cauliflower mosaic virus 35S (CaMV35S) promoter was co-transformed into *P. tometosa* mesophyll protoplasts with a fusion protein (BiP:RFP) between red fluorescent protein (RFP) and portions of the chaperone binding protein (BiP). BiP is an ER resident and is used widely as an ER marker [57]. Confocal examination revealed that the fluorescent signals of YFP and RFP clearly overlapped in the protoplasts (Fig. 2c). These results suggest that PtoUBC34 is localized to the ER compartment.

### *UBC34* is expressed ubiquitously and responds to stress factors in poplar

**Fig. 3:**
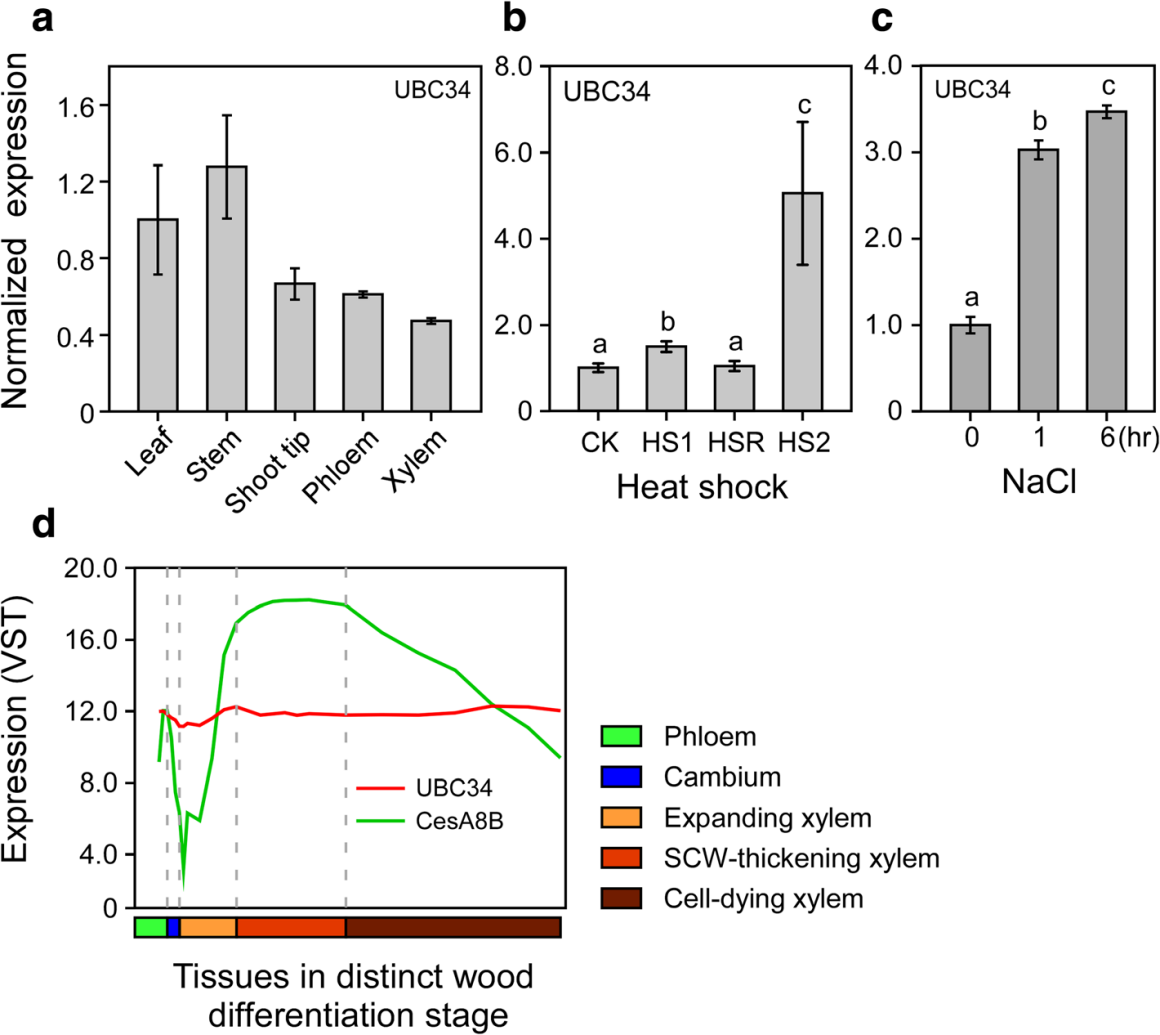
*PtoUBC34* is expressed ubiquitously and responds to stress factors. **a.** qRT-PCR analysis of *PtoUBC34* expression in various poplar tissues, using *Actin6* (Potri.001G309500), *eIF-5A* (Potri.018G107300), and *UBQ* (Potri.014G115100) genes as internal controls, according to Wang et al. [94]. The expression level of *PtoUBC34* in leaves was set as 1. **b.** The expression levels of *PtoUBC34* in mature leaves in response to heat shock treatment by qRT-PCR, using *Actin6* (Potri.001G309500) and *EF1-beta* (Potri.009G018600) genes as internal controls, which were validated in an evaluation assay of eight reference genes during heat shock treatment in *P. tomentosa* (unpublished data). Plants were treated at 37 °C for 1 h (HS1), allowed to recover for 2 h at 24 °C (HSR), and then were subjected to 42 °C for 2.5 h (HS2). The fold-expression was normalized relative to the 24 °C control (CK). **c.** The expression levels of *PtoUBC34* in mature leaves in response to 300 mM sodium chloride (NaCl) treatment were analyzed by qRT-PCR, using *UBQ* (Potri.014G115100) and *TUB* (Potri.003G126800) genes as internal controls, which were validated in an evaluation assay of eight reference genes during salt stress in *P. tomentosa* (unpublished data). The fold-expression was normalized relative to the non-NaCl-treatment control. Data are mean of three biological samples from three plants, respectively, with three technical replicates for each one. Error bar represents standard deviation of the three biological samples. Significance was tested with one-way ANOVA to evaluate the effect of heat shock and NaCl treatment on *UBC34* expression. The different alphabets above the bar indicates statistically significant differences with *p* value < 0.05, while the same alphabet mean no significant differences. **d.** *UBC34* from *P. trichocarpa* was expressed constitutively in distinct differentiation stages across wood formation. The expression data are derived from the ASPWOOD project (http://aspwood.popgenie.org/aspwood-v3.0/) [58]. Phloem, cambium, developing xylem, and mature xylem were collected from stems through longitudinal continuous sections using a cryo-microtome. The tissue type was characterized by examining the images of cross-sections during sampling. All of the samples were collected during the current growth year. Poplar *CesA8-B*, specifically expressed during SCW thickening, was used as a reference

We analyzed the expression pattern of *PtoUBC34* by quantitative real-time polymerase chain reaction (qRT-PCR) in *P. tomentosa* and detected *PtoUBC34* transcripts in all examined tissues, including mature leaf, stem, shoot tip, phloem, and developing xylem (Fig. 3a). To investigate the differential expression level of *PtoUBC34* under heat shock, we treated the plant at 37 °C for 1 h, allowed it to recover for 2 h at 24 °C, and then subjected it to 42 °C for 2.5 h. The accumulation of *PtoUBC34* transcripts was significantly higher after initial heat shock treatment at 37 °C and then reversed after a 2-h recovery from the heat shock. The subsequent heat stress at 42 °C also caused significant increase of *PtoUBC34* transcripts in the leaves (Fig. 3b). Furthermore, we found *PtoUBC34* to be induced significantly by salt (NaCl) treatment (Fig. 3c). During wood formation, *UBC34* in *P. trichocarpa* also has been shown to have a high and constitutive expression in all five traditionally recognized distinct differentiation stages, that is, cambial cell division, cell expansion, secondary wall deposition, lignification, and cell death, according to the research by Sundell et al. [58] (Fig. 3d). This result is in contrast to most of the well-characterized genes during wood formation, which generally are expressed at a high level in a specific wood differentiation stage. For example, poplar *CesA8-B*, a marker gene for secondary cell wall (SCW) formation [59], was highly expressed in SCW-thickening xylem (Fig. 3d). These results suggest that poplar UBC34 might play important roles in plant development and physiology.

### Characterization of the interaction between PtoMYB221/PtoMYB156 and PtoUBC34

**Fig. 4:**
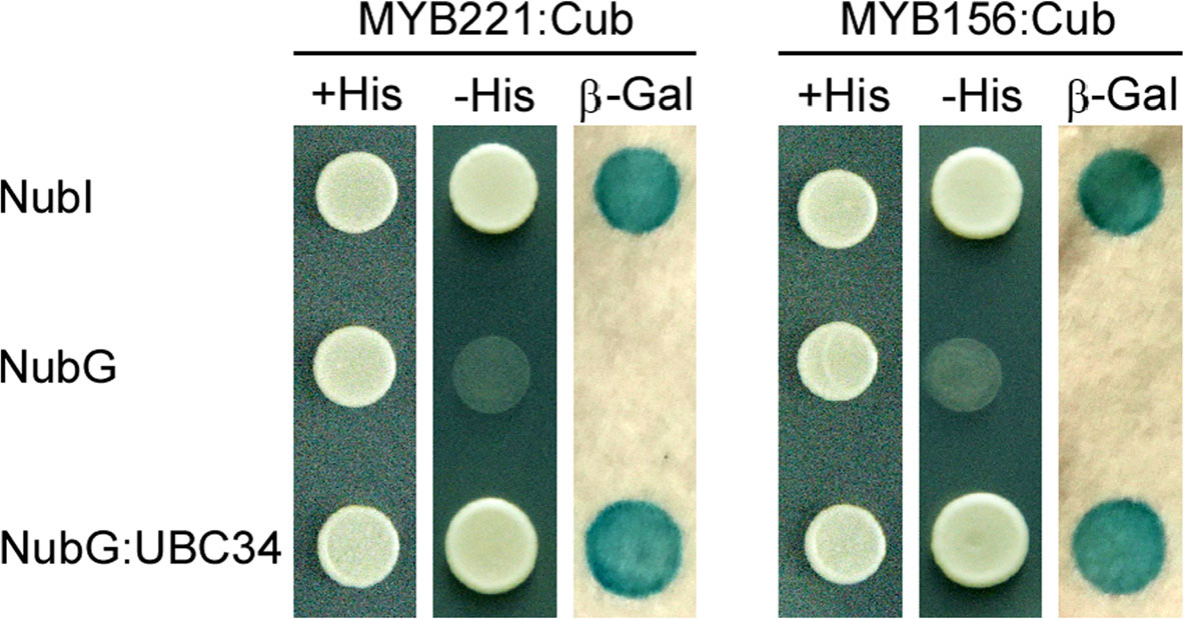
The Y2H demonstrated that full-length PtoUBC34 interacted with both the PtoMYB221 and PtoMYB156 protein in a split-ubiquitin Y2H system. Co-expression with NubI and NubG was used as positive and negative controls, respectively

According to Tuskan et al. [60] and Castanet-Tolosan [13], the poplar genome contains two putative subgroup 4 R2R3-MYB members, MYB221 and MYB156, which are close homologs of *Arabidopsis* MYB4. Analysis by the Y2H assay demonstrated that each of the two subgroup 4 R2R3-MYB members interacted with full-length PtoUBC34 in yeast (Fig. 4).

**Fig. 5:**
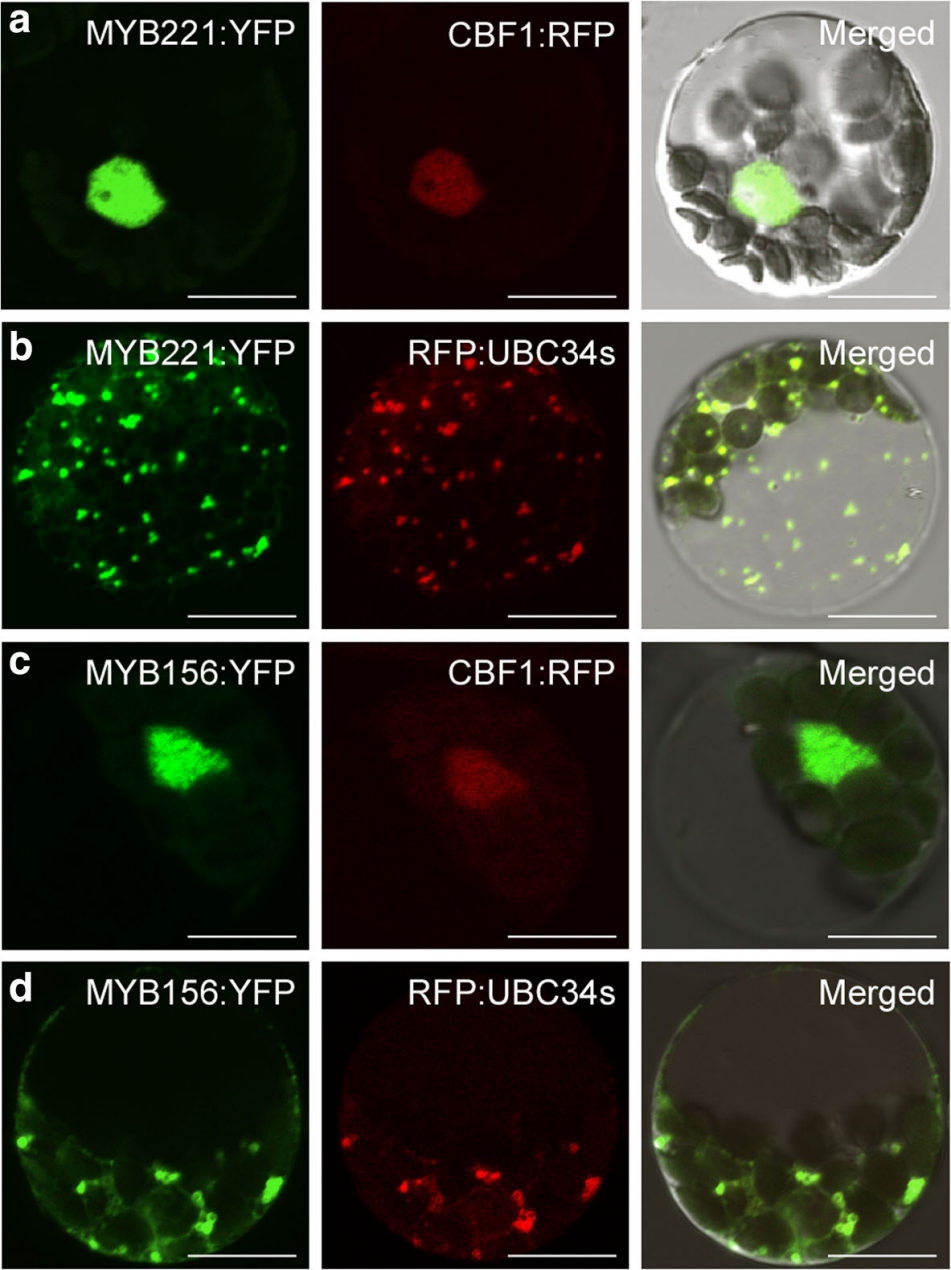
Protein co-localization in poplar mesophyll protoplasts. **a** and **b.** Protein co-localization of PtoUBC34 and PtoMYB221. **c** and **d**. Protein co-localization of PtoUBC34 and PtoMYB156. Both the *PtoMYB221* and *PtoMYB156* were fused with *YFP* and were co-transferred with the nuclear marker *CBF1:RFP* or *RFP:PtoUBC34s* into poplar protoplasts. The co-localization showed that TFs PtoMYB221 and PtoMY156 were translocated from the nucleus (**a** and **c**) to the ER (**b** and **d**) by PtoUBC34. Bar = 10 μm

We performed subcellular co-localization to further investigate the interaction between the proteins. To co-localize each of the subgroup 4 MYB members with PtoUBC34, fusion gene construct either 35S-*PtoMYB221*:*YFP* or 35S-*PtoMYB156*:*YFP* was co-expressed with 35S-*RFP*:*PtoUBC34* in poplar mesophyll protoplasts. Because both 35S-*PtoMYB221*:*YFP* and 35S-*PtoMYB156*:*YFP* vectors showed very weak fluorescent signals in the ER and the degradation of PtoMYB221 and PtoMYB156 protein was suggested under the presence of entire PtoUBC34 protein in plant cells, the PtoUBC34s fused with RFP was used for the subcellular co-localization. Importantly, in the presence of PtoUBC34s, TFs PtoMYB221 and PtoMYB156 were translocated from the nucleus (Fig. 5a and c) to the ER, showing significant overlap with PtoUBC34s and a typical punctate pattern indicative of the ER (Fig. 5b and d). This translocation of PtoMYB221 or PtoMYB156 was exclusive to all co-transfected protoplasts. Overall, these results suggest that transcriptional repressors PtoMYB221 and PtoMBY156 are retained in the ER through interaction with the ER-localized ubiquitin-conjugating enzyme, PtoUBC34.

**Fig. 6:**
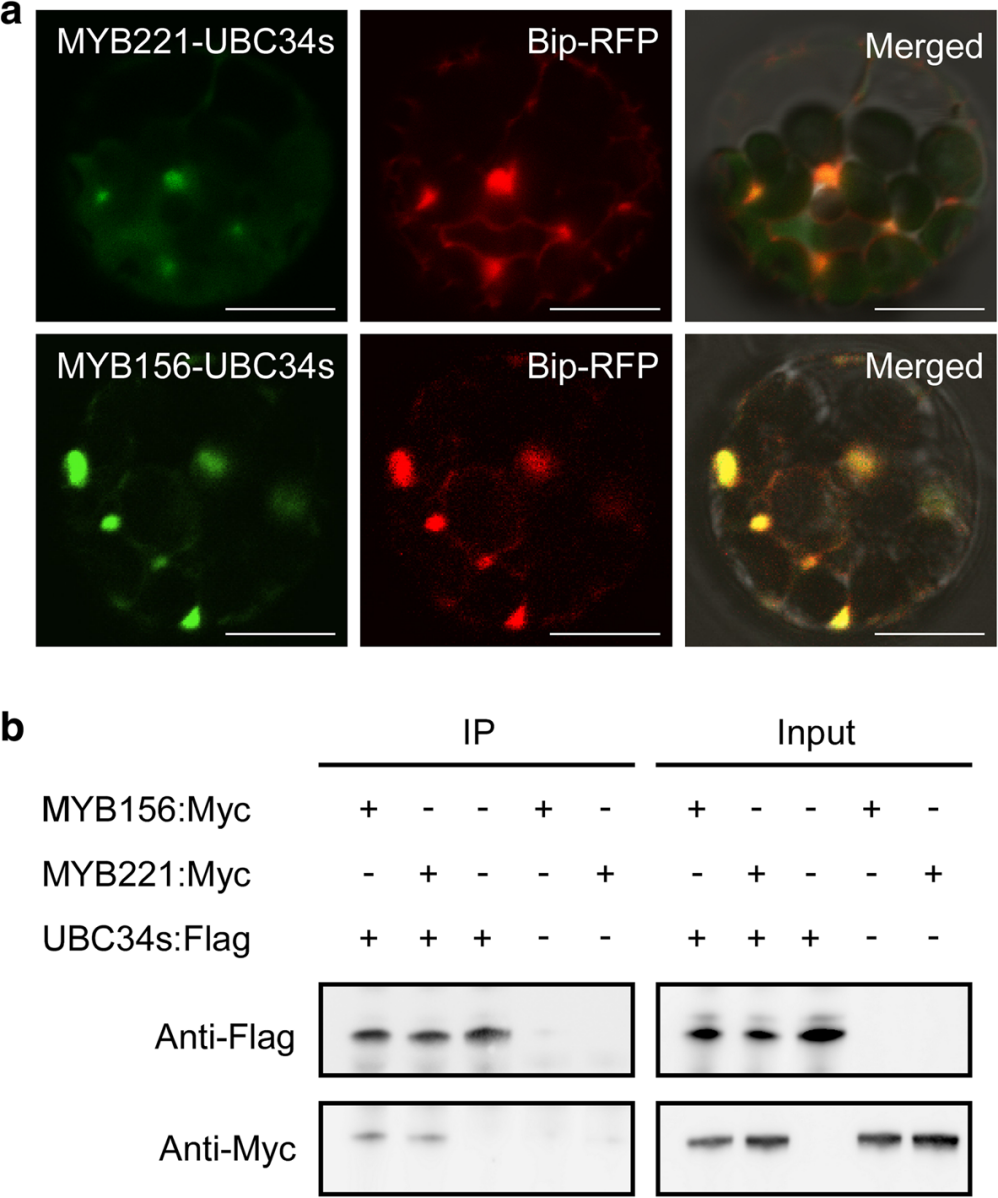
PtoUBC34 interacts with both PtoMYB221 and PtoMYB156 in vivo. **a**. BiFC in poplar protoplasts demonstrated that PtoUBC34 interacts with both the PtoMYB221 and PtoMYB156 in the ER. The BiFC signals co-localized with the marker Bip:RFP in the ER, suggesting that PtoUBC34 and each of PtoMYB221 and PtoMYB156 were co-localized in the same BiFC complex in the ER. Bar = 10 μm. **b**. Coimmunoprecipitation analysis showed an interaction of PtoUBC34s with both the PtoMYB221 and PtoMYB156. *PtoUBC34s:Flag* was co-expressed with each of *MYB221:Myc* and *MYB156:Myc* in poplar protoplasts. Protein extracts were incubated with anti-Flag coupled agarose. Immunoprecipitates (IP) and input proteins were analyzed by immunoblotting using anti-Flag and anti-Myc antibodies as indicated

The co-localization of PtoUBC34 with either PtoMYB221 or PtoMYB156 in the ER of poplar protoplasts and the interaction in yeast provided strong evidence that the pairs of proteins interact with each other in vivo. To further examine this interaction, we performed BiFC assay in poplar mesophyll protoplasts. Only combinations of the eYFP^N^:PtoUBC34s and PtoMYB221:eYFP^C^ pair and the eYFP^N^:PtoUBC34s and PtoMYB156:eYFP^C^ pair—that is, PtoUBC34 fused at the C-terminus to eYFP^N^ (aa 1–173)—gave a positive BiFC signal. The BiFC signal was shown to be co-localized with the Bip:RFP marker in the ER (Fig. 6a), suggesting that PtoUBC34 interacts with both PtoMYB221 and PtoMYB156 in the ER of the plant cell. Furthermore, coimmunoprecipitation assays showed that PtoUBC34s:Flag coimmunoprecipitated with each of PtoMYB221:Myc and PtoMYB156:Myc in poplar mesophyll protoplasts (Fig. 6b), further confirming the interaction between PtoUBC34 and each of PtoMYB221 and PtoMYB156 in vivo.

### PtoUBC34 reduces the repression activity of PtoMYB221 in a dose-dependent manner

**Fig. 7:**
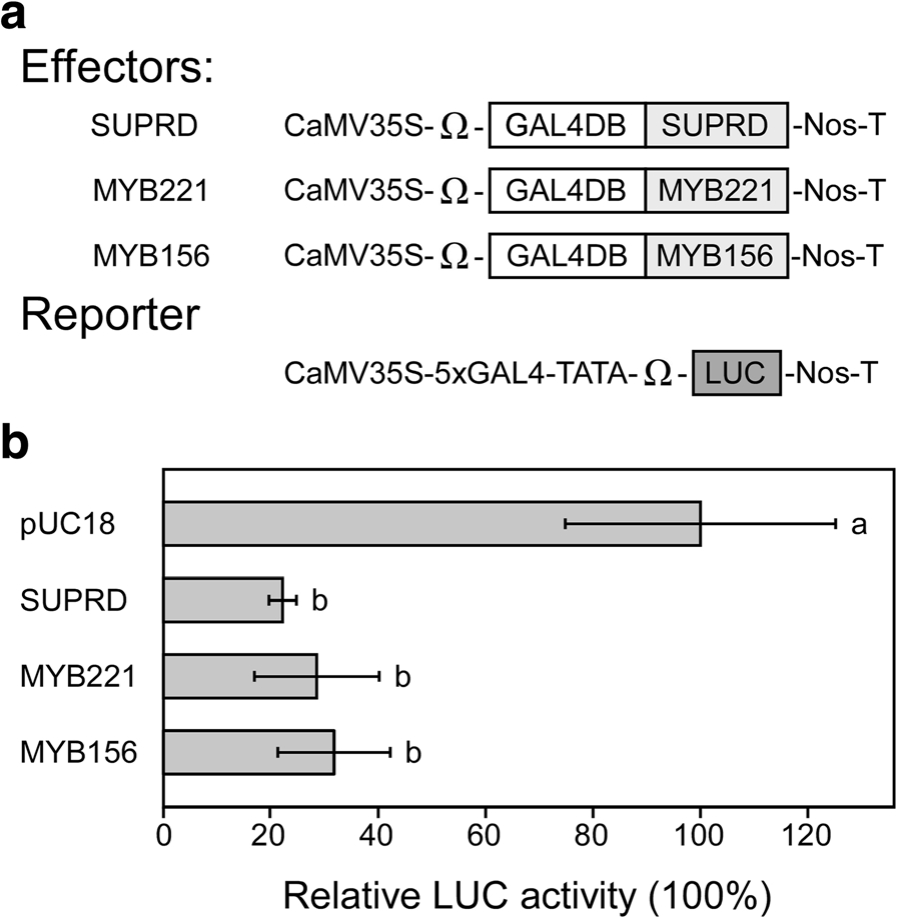
PtoMYB221 and PtoMYB156 are transcriptional repressors. **a.** Schematic representation of the constructs used for evaluation of the repression activity of PtoMYB221 and PtoMYB156 in dual LUC assay in poplar mesophyll protoplasts. The EAR-like motif repression domain of SUPERMAN (SUPRD) was used as a reference repressor [93]. **b.** Relative LUC activities after co-transformation with the reporter and effectors, where pUC18 was used as a control vector. Three independent experiments show similar results. Error bar represents standard deviation of three independent batches of *P. tomentosa* mesophyll protoplast transfections from one of the experiments. Significance was tested with one-way ANOVA to evaluate the roles of the effectors on the *LUC* expression. The different alphabets above the bar indicates statistically significant differences with *p* value < 0.05, while the same alphabet mean no significant differences

**Fig. 8:**
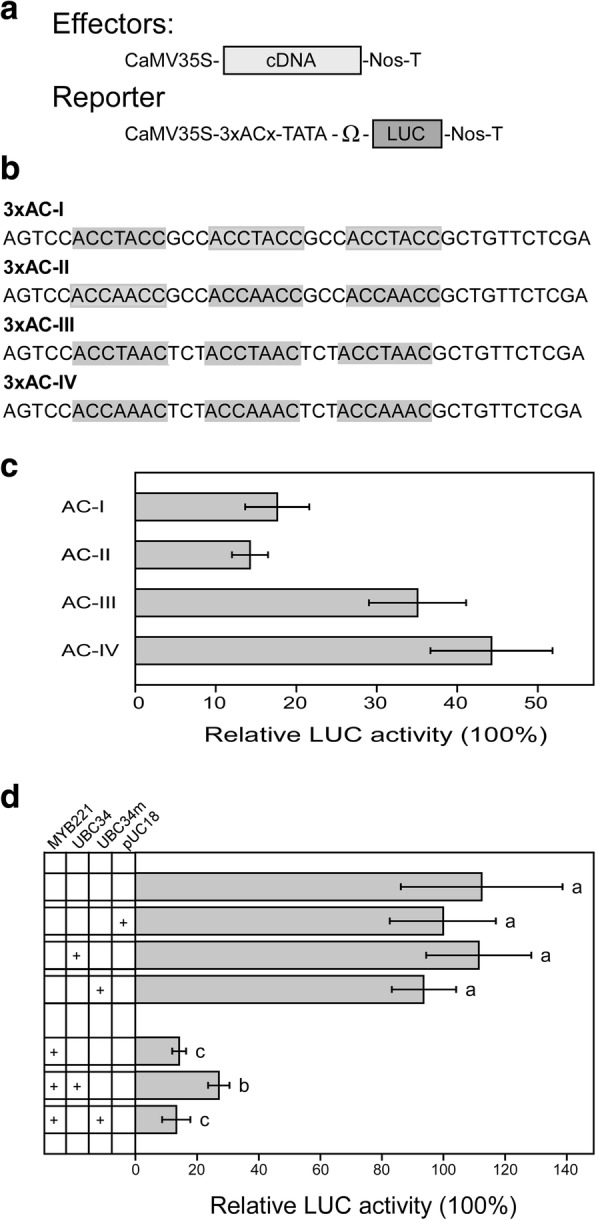
PtoMYB221 targets to the AC *cis-*elements of monolignol pathway genes and its repression activity is reduced with the involvement of PtoUBC34. **a.** Schematic representation of the effector and reporter plasmids used for evaluation of the affinity of PtoMYB221 to AC *cis-*elements in a dual LUC assay. **b.** Synthetic AC elements tested in dual LUC assay. **c.** LUC activity assay driven by PtoMYB221 binding to the AC elements in poplar mesophyll protoplasts. AC-I, AC-II, AC-III, and AC-IV are the reporters containing three copies of the ACx elements fused to the CaMV35S promoter and *LUC* reporter gene. **d.** PtoUBC34 reduced the repression activity of PtoMYB221. 2.4 μg of the effector plasmids PtoMYB221, 600 ng of PtoUBC34 or PtoUBC34m, along with the reporter plasmid and internal control plasmid, were used for co-transfection. Three independent experiments show similar results. Error bar represents standard deviation of three independent batches of *P. tomentosa* mesophyll protoplast transfections from one of the experiments. Significance was tested with one-way ANOVA to evaluate the roles of the effectors on the *LUC* expression. The different alphabets above the bar indicates statistically significant differences with *p* value < 0.05, while the same alphabet mean no significant differences

Both poplar MYB221 and MYB156 members from subfamily 4 of R2-R3 MYB proteins displayed activity of transcriptional repression, which significantly decreased the level of expression of the reporter gene (Fig. 7a and b) and functioned in the transcriptional downregulation of phenylpropanoid and lignin genes [36, 37]. By using electrophoretic mobility shift assay, PdMYB221 was found to bind to the promoter regions of the cell wall biosynthetic genes *PdCESA8*, *PdGT47C*, and *PdCOMT2*, which contain AC element consensus sequences ACC(A/T)A(C/A) C [36]. Therefore, the possible AC elements for PtoMYB221 can be each of the following: ACCTACC (AC-I), ACCAACC (AC-II), ACCTAAC (AC-III), and ACCAAAC (AC-IV), which also are target *cis*-elements for several other subfamily 4 MYB transcriptional repressors [30, 61, 62]. To identify the specific AC element that has the highest affinity for PtoMYB221, we set up a transient expression assay in poplar mesophyll protoplasts in which the reporter plasmid containing the luciferase (*LUC*) reporter gene under the control of CaMV35S promoter with three copies of each of the four AC elements inserted immediately upstream of the TATA box (CaMV35S-3xACx-TATA-*LUC*-NOS), and the effector plasmid encoding the PtoMYB221 protein (CaMV35S-*PtoMYB221*) was co-transfected into protoplasts (Fig. 8a and b). As shown in Fig. 8c, PtoMYB221 effectively repressed the expression of *LUC* reporter gene driven by each of the four AC-I, AC-II, AC-III, and AC-IV elements fused to the CaMV35S promoter with a reduction ranging from 56 to 86% relative to the corresponding control, indicating that PtoMYB221 can bind to all four AC elements. Moreover, PtoMYB221 blocked the expression of the *LUC* reporter gene most effectively (approximately 86%) with the AC-II element, suggesting that PtoMYB221 binds to the AC-II element with the highest affinity.

To analyze the in vivo function of PtoUBC34 on the transcriptional repression activity of PtoMYB221, we co-transformed a *LUC* reporter gene driven by three copies of the AC-II element fused to the CaMV35S promoter and effector genes under the control of CaMV35S promoter in poplar mesophyll protoplasts. The CaMV35S promoter with the AC-II element used here allowed the *LUC* reporter gene to reach a detectable expression level in the presence of the repressor PtoMYB221, which the tissue-specific promoters of lignin biosynthetic genes could not achieve. The effectors included PtoUBC34m in addition to PtoMYB221 and PtoUBC34. PtoUBC34m is a mutated version of PtoUBC34, in which the conserved active site cysteine residue at position 87 is replaced by an alanine residue. This mutant was expected to abolish the ubiquitin-conjugating activity of PtoUBC34 and its various biological functions, as reported in a yeast E2 ubiquitin-conjugating enzyme RAD6 and Pas2 [63, 64], as well as in *Arabidopsis* E2s UBC1 and UBC18 [65, 66]. As shown in Fig. 8d, the expression level of the reporter gene was not significantly affected by co-expression with PtoUBC34, PtoUBC34m, or the control vector pUC18, indicating that the expression of PtoUBC34 and PtoUBC34m did not change the general transcription. Co-expression of the effector PtoUBC34 with PtoMYB221 led to a significant increase in the *LUC* reporter gene activity compared with PtoMYB221 alone as an effector, whereas co-expression of PtoUBC34m with PtoMYB221 did not affect the LUC activity significantly. Because PtoUBC34 did not affect the basal LUC activity, this result suggests that PtoUBC34 reduces the repression activity of PtoMYB221 and likely is involved in the degradation of PtoMYB221.

**Fig. 9:**
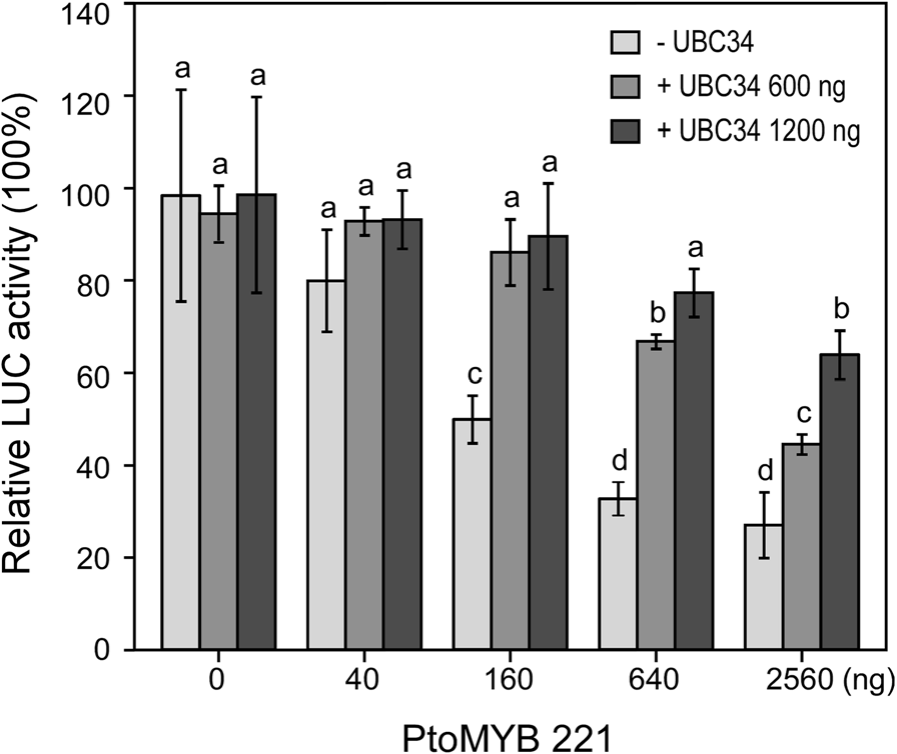
PtoUBC34 reduced the repression activity of PtoMYB221 in a dose-dependent manner. All LUC activities are expressed relative to values obtained after transformation with only the reporter plasmid, which was set at 100%. Three independent experiments show similar results. Error bar represents standard deviation of three independent batches of *P. tomentosa* mesophyll protoplast transfections from one of the experiments. Significance was tested with one-way ANOVA to evaluate the roles of the effectors on the expression level of *LUC* reporter gene. The different alphabets above the bar indicates statistically significant differences with *p* value < 0.05, while the same alphabet mean no significant differences

One of our most significant findings regarding repression activity of PtoMYB221 regulated via PtoUBC34 was that the repression activity depends both on PtoMYB221 and PtoUBC34 protein abundance (Fig. 9). With the increased amount of PtoMYB221, the reporter LUC activity decreased significantly in a dose-dependent manner (Fig. 9). As predicted, the combined transient expression of PtoMYB221 and PtoUBC34 led to a significant decrease in the repression activity of PtoMYB221, compared with that of PtoMYB221 alone as the effector, due to the probable degradation of PtoMYB221 mediated by PtoUBC34 (Fig. 9). Moreover, we observed a lower level of repression activity in the co-transformation with a higher level of PtoUBC34. For example, 2.56 μg PtoMYB221 of the effector plasmid resulted in a 35.16% decrease in the LUC activity compared with the control (without PtoMYB221) in the presence of 1200 ng PtoUBC34, whereas it resulted in a 54.81% decrease with 600 ng PtoUBC34, and a 72.66% decrease when no exogenous PtoUBC34 was added. Indeed, the differential expression of both *PtoMYB221* and *PtoUBC34* might have occurred under the physiological conditions, as heat shock and high salinity significantly induced the transcription of *PtoUBC34* (Fig. 3b and c), and moreover, high temperature, ultraviolet radiation, and cold and high salinity altered the transcriptional expression level of *PtoMYB221* (unpublished data). Overall, these data suggest that the dose-dependent changes of the repression activity of PtoMYB221 on both PtoMYB221 and PtoUBC34 protein abundance might play a physiological role in regulating the transcription of downstream target genes in vivo.

## Discussion

Lignin biosynthesis responds to many developmental and environmental cues, including plant hormones, light, sugar content, circadian rhythms, wounding, and pathogen attacks. To fine-tune the response to the various endogenous and exogenous signals, plants have developed a complex mechanism involving transcriptional hierarchical regulation of lignin pathway genes, which integrates many transcriptional activators and repressors [8, 13]. The repressors play equally important roles as activators in the transcriptional control of lignin biosynthesis, which is regulated by a variety of environmental cues and upstream TFs [8, 20, 46, 67]. In this study, we discovered another layer of the post-translational regulation of the subgroup 4 repressors of the R2R3-MYB family by an ER-localized ubiquitin-conjugating enzyme PtoUBC34 in poplar.

We reported several distinct mechanisms of regulation of the subgroup 4 repressors of the R2R3-MYB family by the ER-localized E2 PtoUBC34 in poplar. We showed that transcriptional repressors PtoMYB221 and PtoMYB156 interact with PtoUBC34 (Figs. 5 and 6), which decreases transrepression activity of PtoMYB221 (Figs. 8d and 9), through either the ERAD of the transcriptional repressors or sequestration in the ER. Typically, the ERAD process, as the primary means of quality control within the protein secretory pathway, targets misfolded or misassembled proteins [51, 53, 68–71]. However, the PtoUBC34-mediated regulation is likely linked with the quantity control of the substrate protein rather than quality control and is independent of the maturation status of substrate proteins. The transient expression assay in poplar mesophyll protoplasts indicated that the increased level of *PtoMYB221* (from 640 ng to 2560 ng per transfection) failed to further reduce the reporter LUC activity, although no exogenous PtoUBC34 was added, suggesting that the native PtoUBC34 was involved in regulating the activity of PtoMYB221 in the poplar protoplasts (Fig. 9). Our conclusion of protein quantity control by the ER-localized E2 PtoUBC34 is supported by the best studied example of the protein abundance control of HMG-CoA-reductase (HMGR) by gp78 ubiquitin ligase complex [72, 73]. Moreover, *PtoUBC34* decreased the transrepression activity of PtoMYB221 in a dose-dependent manner (Fig. 9), and lower transrepression activity of PtoMYB221 occurred with higher levels of *PtoUBC34*. This result is consistent with the data obtained in yeast and *Arabidopsis* in which case the elevated expression level of ERAD components increased the degradation rate of their substrates [50, 52, 74]. This strategy to regulate MYB protein abundance might have physiological significance in plants because the abundance of MYB TFs highly regulates their transactivation activity and the downstream target selectivity [46, 75].

Although PtoMYB221 and PtoMYB156 localize exclusively to the nucleus as a common TF (Fig. 5a and c), its complex with PtoUBC34 is ER specific (Fig. 5b and d). The reduced transrepression activity of MYB221 in protoplast when co-transfected with PtoUBC34 (Figs. 8d and 9) revealed that PtoUBC34 functioned to regulate the transrepression activity of MYB221 by redirecting MYB221 to ER instead of to the nucleus after the TF departed from ribosomal machinery. Whether or not the MYB221 protein is to be degraded in the ER needs further clarification. The interaction between PtoMYB34 and TF PtoMYB221 must occur in the cytosolic side of the ER membrane, in which PtoUBC34 might function in a similar manner as its yeast ortholog Ubc6p with its C-terminal hydrophobic tail embedded in the ER membrane and the catalytic domain facing the cytosol [76, 77]. Conversely, yeast TF Matalpha 2, a well-studied ERAD substrate, gained access in situ to the ERAD ubiquitin-protein ligase Doa10, which was transferred and co-localized at the inner nuclear membrane together with the other components of the ubiquitination machinery [78]. This suggests that substrate-enzyme interaction in the ubiquitination mechanism is regulated spatially, which is also a key aspect of the specificity of E2s and E3s [78, 79]. This mechanism provides real-time surveillance for proteins during the entire journey form ribosomes to their final destination. This notion is supported by the ubiquitination of PHOSPHATE TRANSPORTER1, which was regulated by ubiquitin-conjugating enzyme PHO2/UBC24 in post-endoplasmic reticulum [80] and was ubiquitinated by an E3 ubiquitin ligase NLA in plasma membranes [81]. The ER-localized E2 PtoUBC34 described here provides a potential mechanism for the temporal and spatial regulation of TFs in the compartment of ER prior to reaching the nucleus.

Generally, substrate specificity in ubiquitination is conferred by the E3 ubiquitin ligases [82, 83], which is consistent with the fact that there are many E3 component-associated genes (approximately 1300) in *Arabidopsis* genome [40] and only 37 members in the E2 family [84]. However, many E2 proteins have been shown to interact with the substrates and regulate their stability. For example, tobacco UBC2 interacts with NtERF3 and promotes its degradation [85]. *Arabidopsis* PHO2/UBC24 interacts with PHO1 and mediates the degradation of PHO1 to maintain Pi homeostasis [86]. Moreover, *Arabidopsis* UBC1–6, UBC19–20, and UBC22 have been shown to display variable levels of E3-independent ubiquitination activity [84]. In this study, we found that poplar UBC34 interacted with TF PtoMYB221 in the ER and regulated its transrepression activity (Figs. 6 and 8d). Whether or not PtoUBC34 functions on the ubiquitination and degradation of TF PtoMYB221 as a bonafide E2 and/or an E3-independent E2 needs to be further clarified by additional protein stability and ubiquitination assays.

Poplar TFs PtoMYB221 and PtoMYB156 function as transcriptional repressors in the lignin pathway (Fig. 7), leading to downregulation of lignin pathway genes [36, 37]. However, their physiological roles in plants remain unclear. The contradictory facts appear between the gene expression specificity and their biological function. As transcriptional repressors for lignin biosynthesis, both PtoMYB221 and PtoMYB156 are expressed highly in xylem in which lignin biosynthesis predominantly takes place [36, 37].

**Fig. 10:**
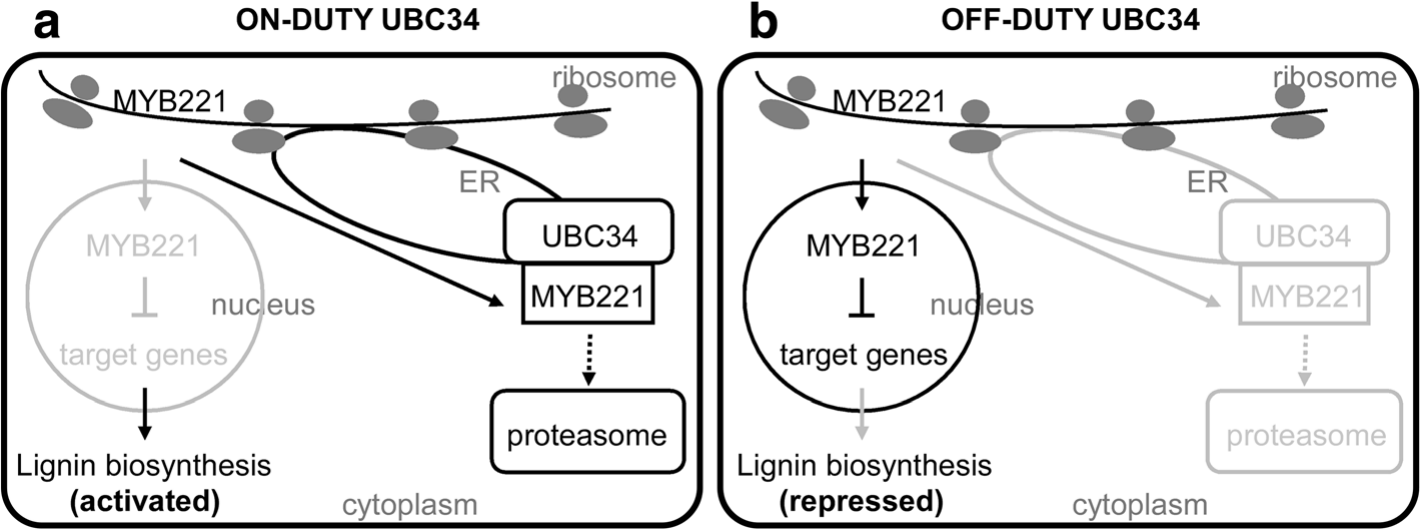
Model of the roles of PtoUBC34 on the regulation of lignin biosynthesis. **a.** Schematic of cell with on-duty UBC34, and the gray part indicated PtoMYB221 absence in nucleus. **b.** Schematic of cell with off-duty UBC34, and the gray part indicated PtoMYB221 absence in ER compartments

We proposed that the activity of PtoMYB221 and PtoMYB156 was fine-tuned by PtoUBC34 and that the translocation of PtoMYB221 and PtoMYB156 to the ER could relieve their repressor activity on phenylpropanoid and lignin pathway. This fine-tuning mechanism might modulate lignin deposition in specific cells under specific physiological conditions by responding to various developmental and environmental cues, such as light, sugar content, circadian rhythms, plant hormones, and wounding (Fig. 10). Further studies will focus on evaluating genetic impact on the transcriptional activity and stability of PtoMYB221 and PtoMYB156 mediated by PtoUBC34 and the influence of environmental cues on the transcriptional activity and stability of the PtoMYB221 protein. This investigation might help to elucidate the fine-tuning mechanism modulating the transcriptional activity of PtoMYB221 and PtoMYB156 and to further elucidate the cross-talk between the lignin biosynthesis pathway and other physiological processes.

## Conclusions

This study has contributed to increase the knowledge about the molecular regulation mechanisms in lignin biosynthesis. To address the role of post-regulation in lignin biosynthesis we screened a Y2H cDNA library of poplar developing vascular tissues against transcriptional repressors PtoMYB221 and PtoMYB156, which was shown to play important roles in lignin biosynthetic pathway. We identified and characterized a *Populus tomentosa* ER-localized E2 ubiquitin-conjugating enzyme, PtoUBC34. We showed that it interacts with transcriptional repressors PtoMYB221 and PtoMYB156. This interaction leads to translocation of PtoMYB221 and PtoMYB156 from the nucleus to the ER and reduces their repression activity in a PtoUBC34 abundance-dependent manner. Further studies are required to evaluate genetic impact on the transcriptional activity and stability of PtoMYB221 and PtoMYB156 mediated by PtoUBC34. Even so, our data here indicate a mechanism by which lignin biosynthesis is regulated by an ER-localized E2 ubiquitin-conjugating enzyme in plants, which probably links the ERAD process with lignin biosynthesis.

## Methods

### Plant materials

*P. tomentosa* ‘BJHR01’ was maintained as described [87]. We used four-week-old plants cultured on MS (Murashig and Skoog) medium with 3% sucrose and 0.6% agar for isolation of mesophyll protoplasts. These plants were multiplied by nodal segments in vitro every 6–8 weeks. We selected five healthy, rapidly growing, two-year-old trees at a field site in Beijing, China, for collection of developing vascular tissues samples for cDNA library construction. The sample collection was done in the morning on a clear day in May 2012. We used a 10 cm region starting from the ground level to collect the differentiating phloem and xylem, and vascular calcium after the stem bark was peeled. All samples were pooled. We used clonally propagated, two-month-old plants grown in a greenhouse under a 16 h/8 h day/night photoperiod at 24 °C for qRT-PCR analysis of the *PtoUBC34* transcript level in various tissues. The following samples were harvested from three well-grown plants: 1 cm segment of shoot tip (shoot tip), the first fully expanded leaf without petiole and main vein (leaf), the stem segment of internode 9 which was undergoing secondary growth (stem), the bottom 10 cm of the stem which was used to collect differentiating xylem and phloem. By peeling off the bark, differentiating xylem was sampled by scraping the surface of the exposed wood, and differentiating phloem was collected by scaping the inner-surface of the exposed bark. The plants that were used for either NaCl or heat shock treatment were subjected to aerated hydroculture with Hoagland nutrient solution in a plant incubator (MLR-352H, Panasonic, Japan) under a 16 h/8 h day/night photoperiod at 24 °C for two months. For heat shock tests, plants were preheated at 37 °C for 1 h and allowed to recover at 24 °C for 2 h prior to 42 °C for 2.5 h. For salt treatments, the medium was supplemented with 300 mM NaCl. Mature leaves with the leaf plastochron index 5 of three plants were harvested either at each time point during the heat shock treatment, or at 0, 1, and 6 h of NaCl treatment. All collected plant materials were flash frozen in liquid nitrogen upon harvesting and stored at − 80 °C until RNA was extracted.

### Library construction and screening

We conducted the Y2H cDNA library construction for *P. tomentosa* developing vascular tissues according to the instructions provided with the normalized cDNA library construction package (#P01011, Dualsystems Biotech AG, Schlieren, Switzerland). The library was screened using PtoMYB221 and PtoMYB156 as the bait in *Saccharomyces cerevisiae* strain NMY51, in accordance with the recommended procedures in the DUALhunter system (#P01601-P01629, Dualsystems Biotech AG, Schlieren, Switzerland). We identified the final isolated positive clones by sequencing.

### qRT-PCR and RT-PCR

We isolated total RNA from mature leaf, stem, shoot tip, phloem, and xylem using RNeasy Plant Mini Kit (#74903, QIAGEN Inc., Valencia, CA, USA) and treated it with DNase I (#79254, QIAGEN Inc., Valencia, CA, USA) to remove DNA. We performed qTR-PCR as previously described [87] using specific primers (Additional file 2: Table S1) with three biological replicates (three biological samples from three plants, respectively). Each reaction was repeated three times. The full-length CDS of *PtoUBC34* was amplified using a primer set *PtoUBC34*-fF1/−fR1(Additional file 2: Table S1).

### Y2H assay and constructs

We performed split-ubiquitin Y2H assay according to the instructions provided in the DUALhunter starter kit (#P01601-P01629, Dualsystems Biotech AG, Schlieren, Switzerland) [48]. The coding sequences of *PtoMYB221* and *PtoMYB156* were amplified using primer sets *PtoMYB221*-yF1/−yR1, *PtoMYB156*-yF1/−yR1 (Additional file 2: Table S1), respectively, and then were cloned into the two *Sfi*I sites of the bait vector pDHB1 and fused at the N-terminus to the Cub fragment. The full-length *PtoUBC34* coding sequence was amplified with primer set *PtoUBC34*-yF1/−yR1, then cloned into the two *Sfi*I sites of the prey vector pPR3-N, and fused at the C-terminus to Nub fragment. Yeast strain NMY51 cells were co-transformed with the resulting constructs and plated onto synthetic medium lacking Leu, Trp, and His. We confirmed the specificity of protein-protein interactions by the activity of β-Gal.

### *P. tomentosa* protoplast isolation and transfection

We isolated *P. tomentosa* mesophyll protoplasts from fully extended leaves of the four-week-old plants grown on MS medium and transfected them with plasmid DNA as described by Guo et al. [88].

### Subcellular localization and co-localization

To construct the fusion proteins YFP:PtoUBC34, C-repeat-binding factor 1:RFP (CBF1:RFP) [89], RFP:PtoUBC34, and RFP:PtoUBC34s, the coding regions of the fluorescent protein (YFP or RFP) and the target protein (PtoUBC34, PtoUBC34s, or CBF1) were amplified using the corresponding primer sets shown in Additional file 2: Table S1, and then were cloned into linearized pGreen0029-35S [90] digested with *Hin*dIII and *Sac*I by homologous recombination, as described (Trelief™ SoSoo Cloning Kit Ver.2, Beijing TsingKe Biotech Co., Ltd.), producing pGreen0029-35S-*YFP*:*PtoUBC34*, pGreen0029-35S-*RFP*:*PtoUBC34*, pGreen0029-35S-*RFP*:*PtoUBC34s*, and the nuclear marker vector pGreen0029-35S-*CBF1*:*RFP*. To prepare YFP-tagged PtoMYB221 and PtoMYB156 protein, the coding region of *YFP* was amplified using primer sets *YFP*-F2/−R2, with a *Bam*HI restriction site included in the forward primer and a *Sac*I site in the reverse primer (Additional file 2: Table S1), cloned into pGreen0029-35S, producing pGreen0029-35S-*YFP*. Then we cloned the polymerase chain reaction (PCR) -amplified product of *PtoMYB221* and *PtoMYB156* coding regions with primer sets *PtoMYB221*-F1/−R1 and *PtoMYB156*-F1/−R1 into the *Kpn*I and *Bam*HI sites of pGreen0029-35S-*YFP*, producing pGreen0029-35S-*PtoMYB221*:*YFP* and pGreen0029-35S-*PtoMYB156*:*YFP*. We prepared the ER marker vector pGreen0029-35S-*Bip*:*RFP* according to the description by Kim et al. [57]. For subcellular localization, *YFP:PtoUBC34* fusion was co-transformed with ER marker *Bip:RFP*, and each of the *PtoMYB221:YFP* and *PtoMYB156:YFP* was co-transformed with nuclear marker *CBF1:RFP* into poplar mesophyll protoplasts. For co-localization, each of the *PtoMYB221:YFP* and *PtoMYB156: YFP* was co-transformed with either *RFP:PtoUBC34* or *RFP:PtoUBC34s* into the mesophyll protoplasts. After incubation for 12 h, we observed the fluorescence under a Nikon inverted fluorescence microscope TE2000-E equipped with a Nikon D-Eclipse A1 spectral confocal laser scanning system (Nikon, Tokyo, Japan). The excitation wavelength was 488 nm for YFP and 543 nm for RFP. We selected the images under a confocal microscope to be green for YFP and red for RFP. When these two fluorescent signals overlapped, the resulting merged image was yellow.

### BiFC

The coding regions of *PtoMYB221*, *PtoMYB156*, and *PtoUBC34s* were amplified using primer sets *PtoMYB221*-F2/−R2, *PtoMYB156*-F2/−R2, and *PtoUBC34s*-F2/−R2, respectively, with a *Bam*HI restriction site included in the forward primer and a *Sma*I site in the reverse primer, and then cloned into pUC-pSPYCE(M) [91], resulting in pUC-35S-*PtoMYB221*:*eYFP*^*C*^, pUC-35S-*PtoMYB156*:*eYFP*^*C*^, and pUC-35S-*PtoUBC34s*:*eYFP*^*C*^. Similarly, the coding regions of *PtoMYB221*, *PtoMYB156*, and *PtoUBC34s* were amplified using primer sets *PtoMYB221*-F2/−R3, *PtoMYB156*-F2/−R3, and *PtoUBC34s*-F2/−R3, respectively, with introduction of *Bam*HI and *Sma*I restriction sites, and cloned into pUC-pSPYNE(R)173 [91], resulting in pUC-35S-*eYFP*^*N*^:*PtoMYB221*, pUC-35S-*eYFP*^*N*^:*PtoMYB156*, and pUC-35S- *eYFP*^*N*^:*PtoUBC34s*. Each of the four pairs *eYFP*^*N*^*:PtoUBC34s* and *PtoMYB221:eYFP*^*C*^, *eYFP*^*N*^*:PtoUBC34s* and *PtoMYB156:eYFP*^*C*^, *PtoUBC34s:eYFP*^*C*^ and *eYFP*^*N*^*:PtoMYB221*, and *PtoUBC34s:eYFP*^*C*^ and *eYFP*^*N*^*:PtoMYB156* together with *Bip:RFP* were co-transfected into the mesophyll protoplasts. We detected fluorescent signals as described earlier.

### Coimmunoprecipitation experiments in poplar mesophyll protoplasts

Coimmunoprecipitation assays were conducted as described previously [92]. The coding sequence of truncated *PtoUBC34* (*PtoUBC34s*) was cloned into the *pCAMBIA1307-3flag* vector yielding *UBC34s-Flag*. The coding sequence of *PtoMYB156* and *PtoMYB221* was cloned into the *pCAMBIA1307-6myc* vector yielding *MYB156:Myc* and *MYB221:Myc*. The *UBC34s:Flag* vector was co-transformed with *MYB156:Myc* or *MYB221:Myc* in poplar protoplasts. After a 16-h incubation, total proteins were extracted from the protoplasts. The protoplast protein extract was incubated with anti-Flag agarose (Sigma-Aldrich) at 4 °C for 2 h. The immunoprecipitates (IP) and input proteins were detected by immunoblotting with either anti-Myc (Abmart) or anti-Flag (MBL) antibodies.

### Effector-reporter-based gene transactivation assays

The vectors CaMV35S-GAL4-TATA-Ω-*LUC*-Nos and CaMV35S-GAL4DB-SUPRD, gifts from Prof. Masaru Ohme-Takagi (National Institute of Advanced Industrial Technology and Science, Tokyo, Japan) [93], were used directly or modified to construct the corresponding reporter vectors CaMV35S-3xACx-TATA-Ω-*LUC*-Nos and effector constructs CaMV35S-GAL4DB-*PtoMYB221*, CaMV35S-GAL4DB-*PtoMYB156*. In brief, we chemically synthesized each of the 3xAC-I, 3x AC-II, 3x AC-III, and 3x AC-IV *cis-*elements of monolignol pathway genes combined with TATA-Ω sequences, which were flanked by *Hin*dIII and *Sal*I sites. The synthesized fragments were digested with *Hin*dIII and *Sal*I and then inserted into *Hin*dIII and *Sal*I- linearized CaMV35S-GAL4-TATA-Ω-*LUC*-Nos, in which the elements GAL4-TATA-Ω were removed, resulting in four reporter constructs CaMV35S-3xAC-I-TATA-Ω-*LUC*-Nos, CaMV35S-3XAC-II-TATA-Ω-*LUC*-Nos, CaMV35S-3XAC-III-TATA-Ω-*LUC*-Nos, and CaMV35S-3XAC-IV-TATA-Ω-*LUC*-Nos. To prepare effector constructs shown in Fig. 7a, CaMV35S-GAL4DB-SUPRD was modified by using a Fast Mutagenesis System (TransGen Biotech, Beijing, China), to produce a *Sma*I restriction site before the element SUPRD, which then was linearized by *Sma*I and *Sal*I. Next, we introduced the PCR products of *PtoMYB221* and *PtoMYB156* coding regions obtained with primer sets *MYB221*-F5/−R5 and *PtoMYB156*-F5/−R5 (Additional file 2: Table S1) into the linearized CaMV35S-GAL4DB-SUPRD (in which the element SUPRD was removed) by homologous recombination (Trelief™ SoSoo Cloning Kit Ver.2, Beijing TsingKe Biotech Co., Ltd.), to obtain effector constructs CaMV35S-GAL4DB-*PtoMYB221* and CaMV35S-GAL4DB-*PtoMYB156*. To prepare effector constructs shown in Fig. 8a, PCR-amplified products of *PtoMYB221* and *PtoMYB156* coding regions with primer sets *PtoMYB221*-F4/−R4 and *PtoMYB156*-F4/−R4 (Additional file 2: Table S1) were cloned into the *Bam*HI-*Eco*RI sites of pGreen0029-35S [90], generating pGreen0029-35S-*PtoMYB221* and pGreen0029-35S-*PtoMYB156*. Mutated PtoUBC34 (PtoUBC34m), in which the cysteine residue at position 87 was replaced with an alanine residue, was made by a Fast Mutagenesis System (TransGen Biotech, Beijing, China). We obtained effector plasmids pGreen0029-35S-*PtoUBC34* and pGreen0029-35S-*PtoUBC34m* by homologous recombination of PCR products of *PtoUBC34* and *PtoUBC34m* coding regions with primer sets *PtoUBC34*-F3/−R (Additional file 2: Table S1) into the *Hin*dIII and *Sac*I sites of linearized pGreen0029-35S [90]. *Renilla* luciferase gene (*RLuc*) under control of the CaMV35S promoter was used as an internal control for normalization of LUC expression values for each transfection. 3.2 μg of each of the reporter constructs was co-transformed with 0.8 μg of *RLuc* internal control construct and corresponding effector constructs into *P. tomentosa* mesophyll protoplasts [88]. After incubation for 12 h, we performed LUC assays with the Dual-Luciferase Reporter Assay System (#E1980, Promega, Madison, WI, USA) and a luminescence reader (GloMax®20/20; Promega, Madison, WI, USA). The LUC expression values of each transfection were normalized to RLuc values. We used pUC18 as the control vector, and expressed all LUC activities relative to values obtained after co-transformation with the reporter plasmid and pUC18 (with the value set at 100%). The experiments were repeated three times. We expressed the values of relative LUC activity as an average of three independent batches of *P. tomentosa* mesophyll protoplast transfections from one of the experiments.

### Accession numbers

The *A. thaliana* and *P. trichocarpa* Genome Initiative locus identifiers or NCBI Protein IDs of the genes investigated in this study are as follows: UBC6 (NP_011026), UBC7 (NP_013735), UBE2J1 (NP_057105), UBE2J2 (NP_477515), UBE2G2 (NP_003334), AtUBC32 (AT3G17000), AtUBC33 (AT5G50430), AtUBC34 (AT1G17280), PtoUBC34 (MH708242), Potri.001G162200, Potri.003G073100, Potri.008G106300, Potri.010G143900, and PeUBC34-like (XP_011048632).

## Additional files

Additional file 1: **Figure S1** Transactivation activity analysis of PtoMYB221 and PtoMYB156 in yeast. (DOCX 146 kb)

Additional file 2: **Table S1** Primer sequences. (DOCX 18 kb)

## Abbreviations

BiFC: Bimolecular fluorescence complementation
Bip: Chaperone binding protein
CaMV35S promoter: Cauliflower mosaic virus 35S promoter
CDS: Coding sequence
Cub: C-terminal half of the ubiquitin
ER: Endoplasmic reticulum
ERAD: ER-associated protein degradation
LUC: Luciferase
Nub: N-terminal half of the ubiquitin
PCR: Polymerase chain reaction
qRT-PCR: Quantitative real-time polymerase chain reaction
RFP: Red fluorescent protein
RLuc: Renilla luciferase
RT-PCR: Reverse transcription polymerase chain reaction
SCW: Secondary cell wall
TF: Transcription factor
UBCc: Ubiqutin-conjugating enzyme catalytic
Y2H: Yeast two-hybridization
YFP: Yellow fluorescent protein
β-Gal: β-galactosidase
