# Supplementary material for: Endoplasmic reticulum–localized UBC34 interaction with lignin repressors MYB221 and MYB156 regulates the transactivity of the transcription factors in Populus tomentosa
Source: BMC Plant Biol. 2019 Mar 12;19:97. doi: 10.1186/s12870-019-1697-y (PMC6416899; doi:10.1186/s12870-019-1697-y)
Supplement: Supplementary file 1 — Figure S1 Transactivation activity analysis of PtoMYB221 and PtoMYB156 in yeast. (DOCX 146 kb) [file 12870_2019_1697_MOESM1_ESM.docx]

**
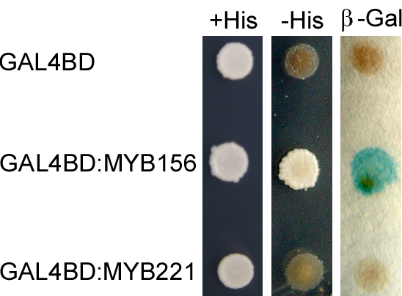
**

**Figure S1.** **Transactivation Activity Analysis of PtoMYB221 and PtoMYB156 in yeast.**

The full-length cDNAs of *PtoMYB221* and *PtoMYB156* were fused in frame with the GAL4 DNA binding domain at the *Nde*I and *Bam*HI sites in the pGBKT7 vector (Clontech), respectively. The constructs were transformed into the yeast strain AH109 containing the *His3* and *LacZ* reporter genes. The transformed yeast cells were grown on SD (synthetic defined) plates with or without histidine and subjected to β-galactosidase (*β-Gal*) activity assay. PtoMYB156 was shown to be able to activate the expression of the *His3* and *β-Gal* reporter genes.
