# Supplementary material for: Endoplasmic reticulum–localized UBC34 interaction with lignin repressors MYB221 and MYB156 regulates the transactivity of the transcription factors in Populus tomentosa
Source: BMC Plant Biol. 2019 Mar 12;19:97. doi: 10.1186/s12870-019-1697-y (PMC6416899; doi:10.1186/s12870-019-1697-y)
Supplement: Supplementary file 2 — Table S1 Primer sequences. (DOCX 18 kb) [file 12870_2019_1697_MOESM2_ESM.docx]

**Table S1. Sequences of primers used in this study**

| **Primer name** | **Sequence (5’ 3’)** | **Use** |
| --- | --- | --- |
| **Primers with homologous recombination sequences used for subcellular localization constructs** | | |
| YFP-F1 | TGGAGAGGACAGCCCAAGCTTATGGTGAGCAAGGGCGAGG | Primer sets YFP-F1/R1 and PtoUBC34-F1/R for **YFP:PtoUBC34**;  RFP-F1/-R1 and CBF1-F/-R for **CBF1:RFP**  RFP-F2/-R2 and PtoUBC34-F2/-R for **RFP:PtoUBC34**;  RFP-F2/-R3 and PtoUBC34s-F1/PtoUBC34-R for **RFP:PtoUBC34s**. |
| YFP-R1 | GATTTCTCAGCCATCTTGTACAGCTCGTCCATGCCGAGAG |  |
| RFP-F1 | CGCTTTGGAGTTACATGGCCTCCTCCGAGAACGTCATCAC |  |
| RFP-R1 | TTCAGCGTACCGAATTCGAGCTCCTACAGGAACAGGTGGT |  |
| RFP-F2 | TGGAGAGGACAGCCCAAGCTTATGGCCTCCTCCGAGAAC |  |
| RFP-R2 | GATTTCTCAGCCATCAGGAACAGGTGGTGGCGGC |  |
| RFP-R3 | GGACGGGCAACAACCAGGAACAGGTGGTGGCGGC |  |
| PtoUBC34-F1 | ACGAGCTGTACAAGATGGCTGAGAAATCGTGTGTCAAGCG |  |
| PtoUBC34-F2 | ACCACCTGTTCCTGATGGCTGAGAAATCGTGTGTCAAGCG |  |
| PtoUBC34s-F1 | ACCACCTGTTCCTGGTTGTTGCCCGTCCTTCTCCAAG |  |
| PtoUBC34-R | TTCAGCGTACCGAATTCGAGCTCTCAAAGTTGAAGCAGAGGCAGC |  |
| CBF1-F | TGGAGAGGACAGCCCAAGCTTATGAACTCATTTTCAGCTTTTTCTG |  |
| CBF1-R | TCGGAGGAGGCCATGTAACTCCAAAGCGACACGTCACCAT |  |
| **Primers with restriction sites used for subcellular localization constructs** | | |
| YFP-F2 | GGATCCATGGTGAGCAAGGGCGAGG | For fusion protein constructs **PtoMYB221:YFP** and **PtoMYB156:YFP** |
| YFP-R2 | GAGCTCCTACTTGTACAGCTCGTCCATGCCGA |  |
| PtoMYB221-F1 | GGTACCATGGGAAGGTCTCCTTGC |  |
| PtoMYB221-R1 | GGATCCTTTCATCTCCAAACCTCTATAATCCAAG |  |
| PtoMYB156-F1 | GGTACCATGGGAAGGTCTCCATG |  |
| PtoMYB156-R1 | GGATCCTTTCATCTCTAAACTTCTATAATCCAAAAC |  |
| **Primers for BiFC constructs** | | |
| PtoMYB221-F2 | GGATCCATGGGAAGGTCTCCTTGC | For BiFC constructs:  **PtoMYB221:eYFP^C^**  **PtoMYB156:eYFP^C^**  **PtoUBC34s:eYFP^C^**  **eYFP^N^:PtoMYB221**  **eYFP^N^:PtoMYB156**  **eYFP^N^:PtoUBC34s** |
| PtoMYB221-R2 | CCCGGGTTTCATCTCCAAACCTCTATAATCCAAG |  |
| PtoMYB156-F2 | GGATCCATGGGAAGGTCTCCATG |  |
| PtoMYB156-R2 | CCCGGGTTTCATCTCTAAACTTCTATAATCCAAAAC |  |
| PtoUBC34s-F2 | GGATCCGTTGTTGCCCGTCCTTC |  |
| PtoUBC34s-R2 | CCCGGGAAGTTGAAGCAGAGGCAGC |  |
| PtoMYB221-R3 | CCCGGGTCATTTCATCTCCAAACCTCTATAATCC |  |
| PtoMYB156-R3 | CCCGGGTTATTTCATCTCTAAACTTCTATAATCCAAA |  |
| PtoUBC34s-R3 | CCCGGGTCAAAGTTGAAGCAGAGGCA |  |
| **Primers for qRT-PCR** | | |
| PtoUBC34-rF | GTACAGGGGTCGATGCCGAC | qRT-PCR |
| PtoUBC34-rR | AGCATTAGAACAACCTGCCGA |  |
| Actin6-rF | AAACTGTAATGGTCCTCCCTCCG |  |
| Actin6-rR | GCATCATCACAATCACTCTCCGA |  |
| eIF-5A-rF | TCGTTCCTTCATCTCACAACTGT |  |
| eIF-5A-rR | AGACTCACAAAGCCATCTTCAGA |  |
| UBQ-rF | CCTTTGTGCTTGATGTGGGC |  |
| UBQ-rR | TAAAGCCAGGAGATGCAGCC |  |
| TUB-rF | AGCTGGCCGTGAACCTAATC |  |
| TUB-rR | CAGCTCAGGGATGGTCAAGG |  |
| EF1-beta-rF | AACCTGGTCGTGATTTCCCT |  |
| EF1-beta-rR | ATCACCAGCAGCCTCCTTG |  |
| **Primers for Effector-Reporter vectors** | | |
| PtoMYB221-F4 | GGATCCATGGGAAGGTCTCCTTGC | Effector-Reporter vectors |
| PtoMYB221-R4 | GAATTCTCATTTCATCTCCAAACCTCTATAATCC |  |
| PtoMYB156-F4 | GGATCCATGGGAAGGTCTCCATG |  |
| PtoMYB156-R4 | GAATTCTTATTTCATCTCTAAACTTCTATAATCCAAA |  |
| PtoUBC34-F3 | GGAGAGGACAGCCCAAGCTT ATGGCTGAGAAATCGTGT |  |
| PtoMYB221-F5 | TCGCCGGTATTGCAATACCCATGGGAAGGTCTCCTTGC |  |
| PtoMYB221-R5 | GGGAAATTCGAGCTCGTCGACTCATTTCATCTCCAAACCTCTATAATCC |  |
| PtoMYB156-F5 | TCGCCGGTATTGCAATACCCATGGGAAGGTCTCCATG |  |
| PtoMYB156-R5 | GGGAAATTCGAGCTCGTCGACTTATTTCATCTCTAAACTTCTATAATCCAAA |  |
| **Primers for Y2H constructs** | | |
| PtoMYB221-yF1 | GGCCATTACGGCCATGGGAAGGTCTCCTTG | Y2H constructs |
| PtoMYB221-yR1 | GGCCGAGGCGGCCAATTTCATCTCCA AACCTCTATAATC |  |
| PtoMYB156-yF1 | GGCCATTACGGCCATGGGAAGGTCTCCATG |  |
| PtoMYB156-yR1 | GGCCGAGGCG GCCAATTTCATCTCTAAACTTCTATAATC |  |
| PtoUBC34-yF1 | GGCCATTACGGCCATGGCTGAGAAATCGTGTGT |  |
| PtoUBC34-yR1 | GGCCGAGGCGGCCTCAAAGTTGAAGCAGAGGCA |  |
| **Primers for RT-PCR to obtain the full-length CDS of PtoUBC34** | | |
| PtoUBC34-fF1 | AACTCTCTCTCTCCCTTGCC | RT-PCR |
| PtoUBC34-fR1 | AAGCATTAGAACAACCTGCC |  |
